# Validation and Application of a Benchtop Cell Sorter in a Biosafety Level 3 Containment Setting

**DOI:** 10.1089/apb.20.0065

**Published:** 2021-11-24

**Authors:** Lydia M. Roberts, Rebecca Anderson, Aaron Carmody, Catharine M. Bosio

**Affiliations:** ^1^ Immunity to Pulmonary Pathogens Section, Laboratory of Bacteriology, Rocky Mountain Laboratories, National Institute of Allergy and Infectious Diseases, National Institutes of Health, Hamilton, Montana, USA.; ^2^ Biorisk Management Branch, Division of Occupational Health and Safety, Office of Research Services, National Institutes of Health, Hamilton, Montana, USA.; ^3^ Research Technologies Branch, Rocky Mountain Laboratories, National Institute of Allergy and Infectious Diseases, National Institutes of Health, Hamilton, Montana, USA.

**Keywords:** biocontainment, biological select agents and toxins, infectious agent, biosafety level 3, pathogen

## Abstract

**Introduction:** Fluorescent-activated cell sorting (FACS) is often the most appropriate technique to obtain pure populations of a cell type of interest for downstream analysis. However, aerosol droplets can be generated during the sort, which poses a biosafety risk when working with samples containing risk group 3 pathogens such as *Francisella tularensis*, *Mycobacterium tuberculosis*, *Yersinia pestis*, and severe acute respiratory syndrome coronavirus 2. For many researchers, placing the equipment required for FACS at biosafety level 3 (BSL-3) is often not possible due to expense, space, or expertise available.

**Methods:** We performed aerosol testing as part of the biosafety evaluation of the MACSQuant Tyto, a completely closed, cartridge-based cell sorter. We also established quality control procedures to routinely evaluate instrument performance.

**Results:** The MACSQuant Tyto does not produce aerosols as part of the sort procedure.

**Discussion:** These data serve as guidance for other facilities with containment laboratories wishing to use the MACSQuant Tyto for cell sorting. Potential users should consult with their Institutional Biosafety Committees to perform in-house risk assessments of this equipment.

**Conclusion:** The MACSQuant Tyto can safely be used on the benchtop to sort samples at BSL-3.

## Introduction

The ability to sort pure populations of immune cells is a critical tool for immunologists. Traditionally, multiparameter, droplet-based cell sorters have been used to isolate cells for downstream analyses (reviewed in Ref.^1^). The utility of these instruments is undeniable, and they have allowed for a plethora of important advances in a wide variety of fields.^2–5^ However, they have inherent limitations. First, there is a level of cell loss that is not easily, if at all, controlled.^6^ This can require the use of additional animals to compensate for loss. Second, depending on the size of the target cell population, the time required to collect that population can be limiting for additional analyses.^1,6^ Lastly, there is the propensity for the generation of aerosols during the sorting process.^7^

Due to the potential of aerosol exposure to the sort operator, a risk assessment should be performed before working with unfixed samples. Holmes et al. provide the current International Society for the Advancement of Cytometry guidelines for sorting samples at all biosafety levels.^8^ There are a number of high-consequence, risk group 3 (RG3) pathogens, such as *Francisella tularensis*, *Mycobacterium tuberculosis*, *Yersinia pestis*, and severe acute respiratory syndrome coronavirus 2, that require manipulation at biosafety level 3 (BSL-3). Handling these organisms at BSL-3 is necessary because one of the primary risks of these pathogens is the ability to cause pulmonary infection following inhalation of aerosols. The risk of aerosolization of infectious material during sorting has been partially mitigated with built-in engineering controls such as the Aerosol Management System (AMS) for the FACSAria II and the placement of cell sorters in biosafety cabinets (BSC).^9^ However, the potential to generate aerosols in combination with the aforementioned challenges remains an insurmountable hurdle for many laboratories in need of sorting cells to a high degree of purity.

Ideally, cell sorting in the BSL-3 laboratory setting should fulfill the following criteria. First, the process should be safe with limited or the complete absence of aerosol generation. Second, the sorting process should be efficient with a reduction or cessation of cell loss. Third, there is the capability to sort using multiple parameters to increase identification of targeted cell populations. Fourth, the sample should remain sterile. Finally, the equipment must be straightforward to use and maintain to facilitate the routine application of cell sorting for immunological studies. Recently, Miltenyi Biotec unveiled a completely closed cell sorting system they coined the MACSQuant Tyto (Tyto). This sorter uses microfluidics to gently sort cells. Other microfluidic cell sorters are on the market, including the On-Chip Sort and NanoCellect Wolf. However, the Tyto best fulfilled all of the requirements described above for potential use in containment settings. Before application it was necessary to validate the absence of the generation of aerosol following a sort as part of our Institutional Biosafety Committee (IBC) risk assessment. Herein, we describe the validation procedures for use of the Tyto in BSL-3 settings. In addition, we provide a quality control method to periodically check instrument performance.

## Materials and Methods

### Aerosol Testing

Internally fluorescent (excitation 480 nm; emission 520 nm) 1.0 μm Dragon Green beads (Bangs Laboratories, Inc.) were diluted 1:100 in Tyto running buffer (Miltenyi Biotec). Aerosol testing was performed on FACSAria II (BD Biosciences) with the Aerosol Management System disabled as a positive control for aerosol generation or MACSQuant Tyto (Tyto; Miltenyi Biotec). Aerosol samples were collected using a Cyclex-d impactor sampling cassette, MegaLite pump, and rotameter (Environmental Monitoring Systems) using a constant vacuum set to 20 L/min. Samples were collected for up to 30 s on the FACSAria II, 10 min with the Tyto door closed, and 30 s with the Tyto door open immediately after the sort completed. Following sample collection, the coverslip was inverted onto a microscope slide and viewed using an Axio Imager (Zeiss).

### Quality Control Testing

Veri-Cells, prestained and fixed lyophilized human peripheral blood mononuclear cells (BioLegend), were reconstituted according to the manufacturer's instructions in 2 mL of Tyto running buffer and then loaded into the sort cartridge. CD4^+^ cells were sorted on the Tyto using R-phycoerythrin as the trigger channel and the VioBlue channel to determine cell speed. Input, sorted, and negative fractions were analyzed on the Symphony flow cytometer (BD Biosciences) and subsequently in FlowJo 10 (BD Biosciences). Singlets were gated by plotting forward scatter height (FSC-H) versus forward scatter area (FSC-A). From the singlet gate, cells were gated by plotting side scatter area (SSC-A) versus FSC-A. Within the cell gate, CD4^+^ cells were gated by plotting CD3 Pacific Blue versus CD4 allophycocyanin. Sort efficiency was calculated as follows: (number of target cells in the sort fraction)/(number of target cells in the sort fraction) + (number of target cells in the negative fraction). Depletion yield was calculated as follows: (percentage of target cells in input fraction – percentage of target cells in the negative fraction)/(percentage of target cells in the input fraction).

## Results

### Absence of Detectable Generation of Aerosols During Use of the Tyto

**Figure 1.**
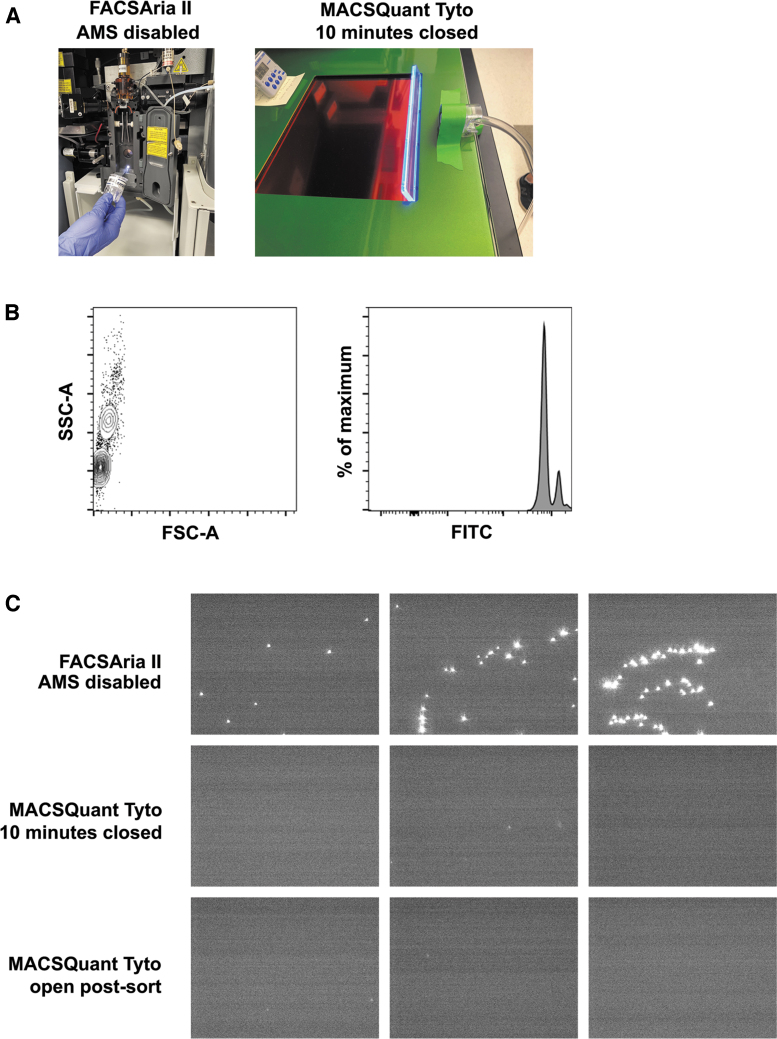
Aerosols are not generated by the Tyto cell sorter. **(A)** Aerosol testing impactor setup on the FACSAria II with AMS disabled and the Tyto. **(B)** A solution containing 1.0 μm internally fluorescent beads in the FITC channel was used for aerosol testing. **(C)** Representative images of coverslips analyzed after aerosol testing on the FACSAria II or Tyto with a closed (during sort) or open (postsort) door. Aerosol testing was performed in triplicate for each condition. The entire coverslip from each Tyto test was scanned and no beads were detected. AMS, aerosol management system; FITC, fluorescein isothiocyanate.

As described above, the generation of aerosols during cell sorting procedures is an important impedance to operation of flow cytometric-based cell sorting in containment settings. Therefore, before usage of the Tyto on the benchtop to sort cells at BSL-3, it was necessary to empirically determine if aerosols were generated during the cell sort procedure. We utilized a novel method described by Perfetto et al.^10^ using internally fluorescent 1.0 μm beads to perform aerosol testing on the Tyto. These beads were uniform in size and intensely fluorescent in the fluorescein isothiocyanate (FITC) channel (Figure 1A). As a positive control, we detected aerosols generated by the in-house FACSAria II when the Aerosol Management System was disabled, and the flow stream disrupted. As expected, beads were detected on the coverslip after only a 30-s exposure (Figure 1B).

During aerosol testing of the Tyto, we recapitulated instrument use during a cell sort. The manufacturer's instructions were followed to prime and load the sort cartridge into the instrument. The Tyto will only sort if the door is closed, and thus, the impactor cassette was placed adjacent to the closed door and allowed to run for 10 min while the fluorescent beads were sorted. If aerosols were generated during the sort procedure, the user could be exposed when the Tyto's door is opened at the sort's completion for removal of the cartridge. Therefore, at the end of the 10 min, the impactor cassette was removed and replaced with a new one before opening the Tyto door. The new impactor cassette was used to sample next to the sort cartridge while it was still in the instrument. We sampled the entire duration the door remained open before it defaulted to closing; the sample time was ∼30 s. All coverslips were visualized using the same gain and focal plane where beads were detected from our positive control. We did observe autofluorescent debris, which clearly differed in size and fluorescent intensity compared to the beads. This observation was not surprising given the amount of air that was passed across the coverslip during the testing procedure. The entire coverslip was scanned; no beads were detected from samples collected when the Tyto door was open or closed (Figure 1B). These data indicate that aerosols were not generated during the cell sort procedure and the Tyto can safely be used on the benchtop at BSL-3.

### Quality Control Testing of Tyto

**Figure 2.**
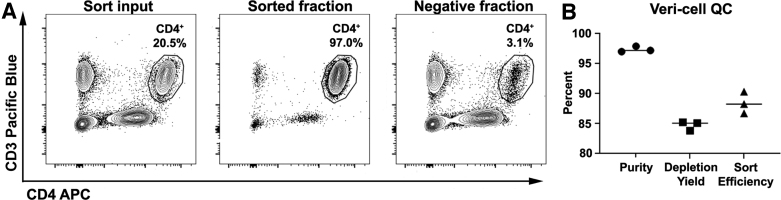
Establishing quality control sort of Veri-Cells. **(A)** Representative flow plots for the Veri-Cell sort input, sorted fraction, and negative fraction showing the percentage of CD4^+^ T cells in each sample analyzed on a BD Symphony flow cytometer using the gating strategy described in the Materials and Methods section. **(B)** The percent purity, depletion yield, and sort efficiency of three independent quality control sorts were calculated.

Routine quality control testing of laboratory equipment ensures consistent instrument performance and can reveal mechanical issues before their use on precious samples. Standard testing procedures also allow for new users to be trained on the equipment with an expected and historically consistent outcome. To this end, we established a quality control sort to periodically verify that the Tyto was performing as expected. We selected lyophilized Veri-Cells as a commercially available, consistent sample to sort cells from using a standardized sample volume, cell concentration, and gating strategy. CD4^+^ T cells were ∼20% of the input sample and were sorted to 97% purity (Figure 2A). Because there is no sample loss when sorting on the Tyto, the negative fraction could also be analyzed to determine the extent of target population depletion (Figure 2A). The depletion yield was >80% and the calculated sort efficiency was >85% (Figure 2B). This quality control analysis was performed multiple times over the six-plus months the instrument has been in use and demonstrates the stability of the Tyto over time in our hands. Furthermore, at least 1 month passed between each quality control analysis and it was often the case the machine was not utilized during this time. Our laboratory has established a purity of >96%, depletion yield of >80%, and sort efficiency of >85% as benchmarks that must be met during quality control testing.

## Discussion

Isolation and downstream analysis of specific immune cell populations has been a critical component of immunological research over the last several decades. Over this same period, droplet-based sorters have constantly evolved to include additional lasers and/or channels, thereby allowing end-users to sort on an increasing number of fluorescent parameters. Although the sorting technology has improved with time, one feature remained consistent. That is, the danger of generating aerosols with infectious potential during the sort procedure. This small but important feature typically precludes the use of droplet-based sorters within containment laboratories. Some groups have circumvented this hurdle by placing the instrument in custom-built, expensive, and large BSCs. However, given their expense and footprint, this is often not an option for most institutes working with RG3 or 4 pathogens. To circumvent this challenge within our own BSL-3 laboratory, we acquired the MACSQuant Tyto (Tyto).

The primary feature of the Tyto that facilitates its benchtop use in a containment setting is the ability to gently and rapidly sort cells within a sterile closed cartridge without generating aerosols. The Tyto uses microfluidics and extremely low air pressure to flow cells past the instrument's lasers. A rapidly moving gate opens to divert a desired cell into the sort fraction and then closes again. This technology does not rely on cells to be within droplets and they are not subjected to any charge changes to deflect them into a sort tube such as traditional droplet-based sorters. Thus, this technology is not only appropriate for containment sorts but is also attractive for use at BSL-1/BSL-2 for cell types that are extremely fragile and susceptive to mechanical perturbations. Although the Tyto may be appropriate for a variety of sorts outside and inside of containment, it should be emphasized that this instrument sorts one population at a time within the closed cartridge. Thus, indexed single cell sorts, sorting into plates, and sorting more than one population at a time are not possible.

Before implementation of the Tyto as a benchtop sorter in BSL-3, it was necessary to confirm that aerosols consistent with those capable of carrying infectious organism were not generated during operation of the equipment. We utilized a recently published and validated method for testing aerosol generation by droplet-based cell sorters to determine the aerosol generating potential of the Tyto.^10^ This testing protocol was straightforward to complete and not cost-prohibitive. We utilized a commercially available vacuum, impactor cassette, and 1.0 μm internally fluorescent beads to determine whether the Tyto generated aerosols during the sort procedure. As a positive control, the in-house FACSAria II was placed in fail mode and clearly visible aerosols were collected using the same setup. Importantly, no aerosols were generated by the Tyto during a sort of fluorescent beads. Overall, the testing procedure took less than 4 h to collect and analyze the samples. Thus, it should be easily implemented by other institutions or groups to test their own instruments using the protocol outlined herein. The Tyto also has additional safety features built in that are important to note during BSL-3 risk assessments, including the inability to sort if there is a power failure, vacuum failure, an open door, or improper seating of the cartridge.

Additional safety features of the Tyto include the absence of fluidics and numerous stop gap measures that result in the machine not running if certain criteria are met. Without fluidics, there are no decontamination procedures necessary to clean the fluidic lines in preparation for the next user, nor is there waste fluid to dispose of like one must deal with for droplet-based sorters. The only waste generated from the sort is the cartridge itself, which can be disposed of in the typical biohazard waste stream. Second, if there is a flaw in the cartridge, that is, a crack that would not allow it to be pressurized or the cartridge is not seated correctly, the instrument will not run. All cartridges undergo quality control testing via air pressurization by the manufacturer to ensure the integrity of the product. Furthermore, the cartridge priming step by the end-user just before sorting would reveal any defects; if there is a crack, the cartridge will not prime. If a user discovers a flaw, the lot and serial numbers are printed on each cartridge and can be reported to the manufacturer. Finally, the door to access the cartridge cannot open while cells are being sorted and can only be opened after a sorting session is complete or intentionally stopped by the user.

In summary, the ability to sort immune cell populations in high-containment (BSL-3/BSL-4) laboratories has historically been hampered by the risk of aerosol generation by droplet-based cell sorters. While these risks can be mitigated by engineering controls such as placing the instrument in a BSC, these are often cost or space prohibitive. As an alternative, we have utilized the Tyto cell sorter in our BSL-3 laboratory. This instrument not only has a small footprint but most importantly does not generate aerosols during the sort procedure. The use of this technology will uplift current immunological research in containment laboratories by allowing a greater number of research groups to isolate cell types involved in the immune response to high-consequence pathogens for downstream applications.
